# Expression Analysis of CB2-GFP BAC Transgenic Mice

**DOI:** 10.1371/journal.pone.0138986

**Published:** 2015-09-25

**Authors:** Anne-Caroline Schmöle, Ramona Lundt, Benjamin Gennequin, Hanna Schrage, Eva Beins, Alexandra Krämer, Till Zimmer, Andreas Limmer, Andreas Zimmer, David-Marian Otte

**Affiliations:** 1 Institute of Molecular Psychiatry, University of Bonn, Bonn, Germany; 2 Clinic for Orthopaedics and Trauma Surgery, University of Bonn, Bonn, Germany; University Hospital Medical Centre, GERMANY

## Abstract

The endocannabinoid system (ECS) is a retrograde messenger system, consisting of lipid signaling molecules that bind to at least two G-protein-coupled receptors, Cannabinoid receptor 1 and 2 (CB1 and 2). As CB2 is primarily expressed on immune cells such as B cells, T cells, macrophages, dendritic cells, and microglia, it is of great interest how CB2 contributes to immune cell development and function in health and disease. Here, understanding the mechanisms of CB2 involvement in immune-cell function as well as the trafficking and regulation of CB2 expressing cells are crucial issues. Up to now, CB2 antibodies produce unclear results, especially those targeting the murine protein. Therefore, we have generated BAC transgenic GFP reporter mice (CB2-GFPTg) to trace CB2 expression *in vitro* and *in situ*. Those mice express GFP under the CB2 promoter and display GFP expression paralleling CB2 expression on the transcript level in spleen, thymus and brain tissue. Furthermore, by using fluorescence techniques we show that the major sources for GFP-CB2 expression are B cells in spleen and blood and microglia in the brain. This novel CB2-GFP transgenic reporter mouse line represents a powerful resource to study CB2 expression in different cell types. Furthermore, it could be used for analyzing CB2-mediated mobilization and trafficking of immune cells as well as studying the fate of recruited immune cells in models of acute and chronic inflammation.

## Introduction

The CB2 receptor was discovered in 1993 by Munro et al. [1] in a study showing that CB2 mRNA was present in splenocytes, but not detectable in the brain. This observation was confirmed by autoradiography studies using the high affinity CB1/CB2 agonist CP55,940, which showed a distinct pattern of labeling in wildtype (WT) mouse brains and a complete absence of detectable binding in CB1 KO mouse brains [2,3], whereas a clear labeling was present in the CB1 KO spleen [3]. Later, studies confirmed the presence of CB2 in cells of the immune system, such as macrophages, natural killer cells, monocytes, neutrophils, and B and T cells [4]. CP55,940 showed strong binding to the spleen of WT, but not in CB2 KO mice [5], indicating that CB2 is the main cannabinoid receptor on immune cells.

Conversely, immunohistochemistry approaches for the study of CB2 protein expression revealed a widespread labeling for CB2 in the uninjured brain [6–8]. Indeed, there is some pharmacological evidence for potential functional effects of CB2 receptors in the brain [9]. However, this evidence does not support the hypothesis reinforced by immunohistochemistry data indicating that CB2 receptors are broadly expressed in neurons in the brain, which is a highly debatable finding [10].

Baek et al. [11] recently examined CB2 immunolabeling using various commercially available CB2 antibodies on uninjured brain tissue of WT and CB2 KO mice. They found that interpretation of immunohistochemistry results with CB2 antibodies in brain tissue should be done with great caution. Validation of CB2 antibodies often failed when knockout control tissue was included.

To address the problem of antibody specificity, we have now generated BAC transgenic mice expressing GFP under the CB2 promoter (CB2-GFPTg).

## Material and Methods

### Animals

Experimental procedures complied with all regulations for animal experimentation in Germany and were approved by the Landesamt für Natur, Umwelt und Verbraucherschutz in Nordrhein-Westfalen, Germany (AZ: 84–02.04.2012.A146, 8.84–02.05.20.11.101).

Mice carrying the CB2Tg^(eGFP FRT)^ transgenic allele, hereafter referred to as CB2-GFPTg, were derived by microinjection of (C57BL/6J X DBA) F1 eggs and subsequently maintained by backcrossing to C57BL/6J stocks.

### Generation of the CB2-GFP BAC Transgene

**Fig 1:**
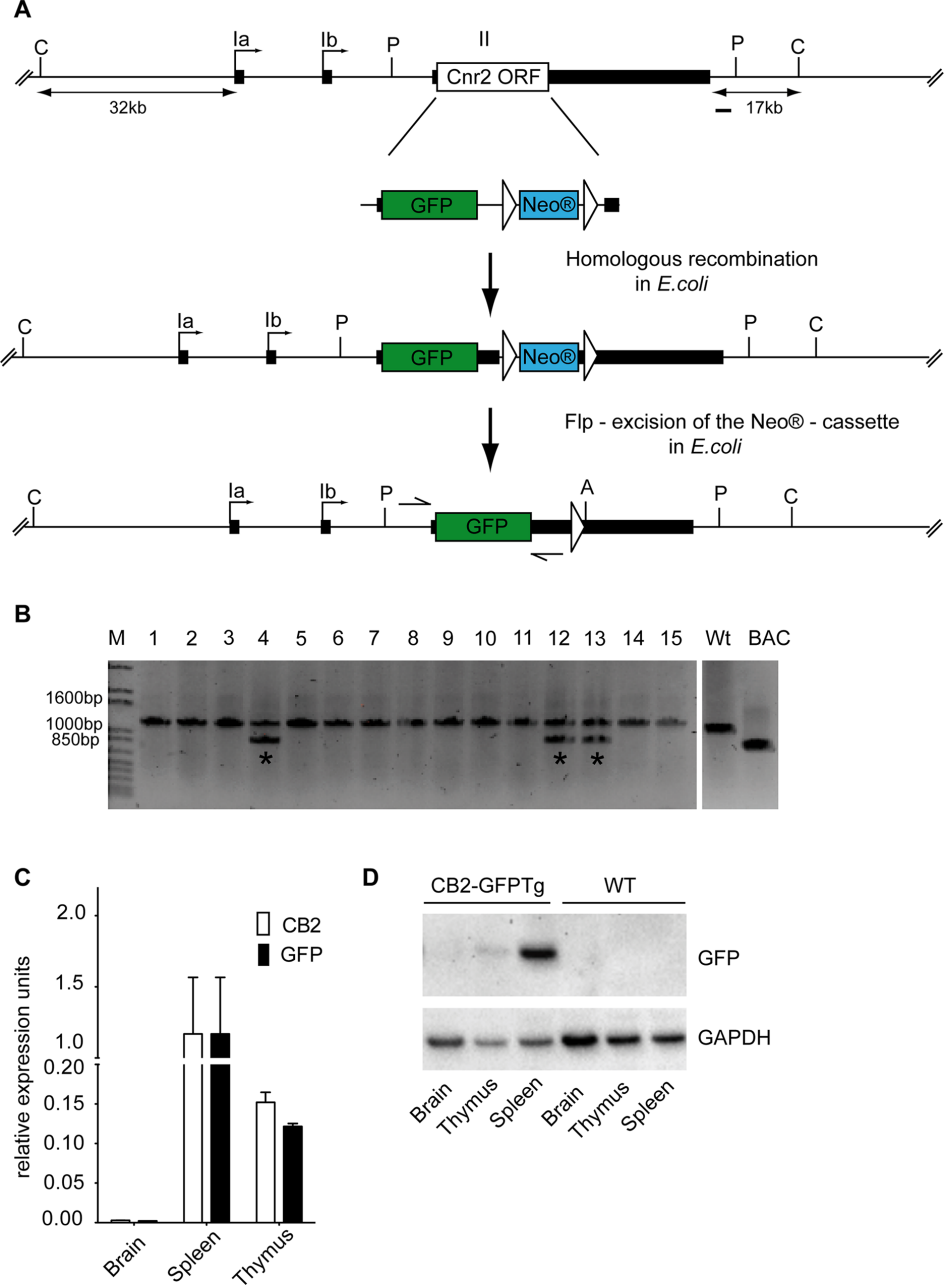
Construction of CB2-GFP BAC transgene and expression in various transgenic mouse tissues. (a) Schematic diagram of GFP-FRT-Neo® BAC modification fragment and homologous recombination into the wild-type Cnr2 BAC depicts Cnr2 exons (black rectangles), ORF (open box), and GFP-FRT-Neo® fusion reporter (green/blue). Cnr2 homology arms (each ∼50 bp) are shown on either side of the insert sequence. Introduction of the GFP-FRT-Neo® cassette leads to a replacement of the Cnr2 ORF without affecting any putative Cnr2 promoter sequences. Excision of the Neo® cassette after integration to derive the final CB2-GFPTg BAC transgene is shown. Restriction enzymes and a genomic probe (black bar) used for *Southern blot* analysis are indicated. Exons are represented as rectangles and FRT sites as triangles. P: *Pst*I; A: *Ase*I; C: *Cla*I. (b) Screening for transgene integration by PCR. Integration of the CB2-GFPTg BAC into the genomic mouse DNA was confirmed by PCR with specific primers (eGFP_Aat_F2 and eGFP_R2) as indicated with arrows in a. Three independent founders were identified (asterisks) by the detection of an additional band representing the GFP. (c) Comparison of GFP and CB2 mRNA expression in brain, spleen and thymus relative to HPRT reference gene expression. In brain tissue samples neither GFP nor CB2 mRNA expression was detectable. Thymus showed moderate expression of both, GFP and CB2, whereas spleen tissue revealed highest expression of GFP and CB2. n = 4. (d) Representative western blot analysis of GFP protein expression in brain, thymus and spleen in CB2-GFPTg and WT mice. CB2-GFPtg mice showed moderate GFP protein expression in thymus and high GFP protein expression in spleen. No detectable GFP protein expression in brain tissue samples as well as in samples of WT littermates. GAPDH was used as loading control.

For the generation of the CB2/eGFP-FRT-Neo® fragment we took advantage of several *in vivo* recombination kits from Gene Bridges. A 9kb fragment from a mouse BAC clone (RP23-3B6, C57BL6/J library) including the CB2 ORF was subcloned from a mouse BAC clone (RP23-3B6, C57BL6/J library), using the primers CB2subF and CB2subR (S1 Table). Next a FRT-PGK-Neo®-FRT PCR fragment was inserted 933bp downstream of the CB2 ORF in the CB2-subclone, using the primers FRTNeoF and FRTNeoR (S1 Table), resulting in the CB2-FRT-Neo®-subclone plasmid (S1 Table). After that, the eGFP ORF from the eGFPN1 (Promega) was amplified using the primers eGFP_F1 and eGFP_Bam_R1 (S1 Fig, S1 Table), to add 50bp genomic mouse sequence adjacent 3´ and 5´ to the CB2 ORF to both sites of the eGFP ORF. Additionally, a cloning *Bam*HI restriction site was inserted at the 3´ end. The primers eGFP_Aat_F2 and eGFP_R2 (S1 Fig, S1 Table) were used to add additional 50 bp genomic mouse sequence to the previously amplified fragment and also a cloning *Aat*II restriction site on the 5´ end. This amplification resulted in a 940 bp fragment comprised of the eGFP ORF flanked up- and downstream by 100 bp mouse genomic sequences and a 3´BamHI and a 5´AatII restriction site. This elongated eGFP PCR product was then ligated and cloned via *Aat*II and *Bam*HI digestion site into the likewise cut CB2-FRT-Neo®-subclone plasmid, resulting in the CB2/eGFP-FRT-Neo® plasmid (S1 Fig). This plasmid now contains the eGFP ORF instead of the CB2 ORF. Then the CB2-GFP-FRT-Neo® was amplified using the primers eGFP_F1 and FRTneoR and the CB2-FRT-Neo®-FRT-subclone as template. In the next step, this PCR product containing the CB2/eGFP-FRT-Neo® reporter moiety was introduced into the original mouse BAC RP23-3B6. In this manner, the Cnr2 ORF was replaced by the GFP gene without interfering with putative promoter sequences of the Cnr2 gene (Fig 1A). In the final step, the FRT-PGK-Neo®-FRT selection cassette was cut out using a flp recombinase (Fig 1A). Appropriate integration of the GFP reporter was confirmed by direct sequencing of the BAC clone in conjunction with restriction enzyme analysis.

### Generation of CB2-GFP BAC transgenic mice

CB2-GFP BAC DNA was digested with *Clal* and a 74kb fragment comprising 32kb upstream to Cnr2 exon Ia and 17kb downstream to Cnr2 exon II (S2A Fig) was microinjected into fertilized mouse eggs (C57BL/6J X DBA) as described in [12]. Transgenic founder were identified by PCR using the primers eGFP_Aat_F2 and eGFP_R2 or by *Southern* blot using a genomic probe generated with the primer probe F and probe R (S2A Fig, S1 Table). The CB2-GFPTg mice were maintained on a hemizygote level and were backcrossed to C57BL6/J for at least five generations. Expression across all lines was screened using tail vein blood and flow cytometric analysis like described in [13].

### Total RNA preparation and RT PCR

Mouse tissues from CB2-GFPTg mice and Wt mice were rapidly dissected after cervical dislocation, snap frozen in isopentane and stored at −80°C. Total RNA was prepared using the Trizol method (Invitrogen, Germany). Five μg RNA and 0.5 μg oligo dT (20) primers (Invitrogen) were heated at 70°C for 4 min, chilled on ice and reverse transcribed in a total volume of 20 μl containing 4 μl first strand buffer (Invitrogen), 2 μl 0.1 M DTT, 1 μl 10 mM dNTPs, 0.5 μl RNase OUT (Invitrogen), and 200 U Superscript II reverse transcriptase (Invitrogen) at 42°C for 50 min.

### Quantitative real-time PCR (qPCR)

Quantitative real-time PCR (qPCR) was performed using a LightCycler® 480 (Roche, Germany) on cDNA samples. The PCR reaction was carried out at 50°C for 2 min, 95°C for 10 min followed by 40 cycles of 95°C for 15 s, then 60°C for 1 min using the TaqMan® Gene Expression Master Mix (Applied Biosystems, USA). TaqMan® primer and probe sets were purchased from Applied Biosystems: Mm00438286_m1 for Cnr2, Mr04097229_mr for GFP and Mm01545399_m1 for hypoxanthine guanine phosphoribosyl transferase (HPRT). HPRT was used to normalize for the amount of sample in a given reaction.

### Western blot analysis

Mouse tissues were homogenized in 10 mM Tris HCl pH 8, 150 mM NaCl, 5 mM EDTA, 1% NP-40, 0.5% sodium deoxycholate, and 0.1% SDS containing Complete Mini protease inhibitor cocktail (Roche). Protein concentration was determined using the BCA Protein Assay (Pierce). 20 μg of the protein extracts were separated by 4–12% SDS-PAGE and probed with goat anti-GFP (ab6673) polyclonal antibody (1:1000; BD, overnight at 4°C) followed by rabbit anti-goat peroxidase-conjugated antibody (1:10,000; Jackson ImmunoResearch, 1 h at room temperature) and then exposed to enhanced chemiluminescence substrate (ECL; Pierce) for 5 minutes. After stripping, the blot was labeled with a mouse anti-GAPDH antibody (Abcam 9484, 1:5000, 2 h at room temperature) followed by a goat anti-mouse peroxidase-conjugated antibody (Jackson Lab, 1:3000, 1 h at room temperature). Blots were incubated with ECL for 5 minutes and analyzed using the ChemiDoc™ MP Imagine System from Bio-Rad and the ImageLab 4.01 software.

### Cryosectioning of mouse tissue

Six week old Wt and CB2-GFPTg mice were anesthetized and perfused intracardially with ice-cold PBS, followed by perfusion with ice-cold 4% PFA. Spleens and brains were removed and fixed with 4% PFA at 4°C over night. Tissue was transferred to 20% sucrose and again incubated over night at 4°C. Afterwards, tissue was embedded in Tissue Tec, and stored at –80°C until cyrosectioning. Cryosection was performed with a Cryostat (CM3050S, Leica) and slices of 12 μm thickness were generated.

### Blood extraction

For analysis of GFP expression by different leukocyte subsets, mice were anesthetized and blood was taken from tail vein. Blood was incubated in erythrocytes-lysis buffer (155 mM NH_4_Cl, 7.3 mM K_2_CO_3_, 0.127 mM EDTA and aqua bidest) for 5 min at room temperature. Erythrocyte-free blood cells were washed twice in PBS containing 2% FCS and subjected to cell surface marker staining for flow cytometric analysis.

### Splenocyte isolation

For cultivation of splenocytes, spleens from six week old Wt and CB2-GFPTg mice were isolated and homogenized with a 100 μm cell strainer. Cells were cultured over night at 5% CO_2_, 37°C in RPMI containing 10% FCS, 1% penicillin/ streptomycin, 1% L-glutamine and 500 μl β-mercaptoethanol on IgM-coated cell culture plates and harvested on the next day for flow cytometric analysis. For stimulation, lipopolysaccharide (LPS) (1 μg/ml) or 5 μM cytosine-phosphate-guanosine (CpG)-DNA were added 16h before harvesting.

### Flow cytometry

Cells were labeled with fluorochrome-coupled anti-mouse antibodies or biotin-coupled anti-mouse antibodies, followed by incubation with streptavidin-fluorochrome coupled secondary reagents. Antibodies (RRID): CD4 (L3T4) (AB_466330), CD19 (AB_657663), CD11b (AB_1582236), CD8a (AB_1272198), CD45 (AB_396774), CD335 (NKp46) (AB_1210743), CD16/32 (AB_312801), B220 (AB_396793), TCRβ (AB_466818). Immunofluorescence of labeled cells was subsequently measured by using a FACS Canto II (BD Bioscience, Heidelberg, Germany), equipped with FACSDiva software. Data analysis was performed using FlowJo software (Tree Star Inc., Ashland, USA).

### Immunofluorescence and LSM

Spleen and brain slices of 12 μm thickness were permeabilized with 0.2% or 0.5% Triton X-100 for 20min or 1h, respectively. Slices were blocked with 3% BSA (spleen) or 5% NDS + 0.5% BSA (brain) for 2h at room temperature. Afterwards, spleen and brain slices were stained with primary antibodies at indicated dilutions (in blocking solution) over night at 4°C. A secondary antibody (diluted 1:500) coupled to either AF647 or Cy3 fluorochrome was incubated for 1h at room temperature. Antibodies: Iba1 (AB_839506) 1:500, CD11c (AB_395059) 1:100, B220 (AB_466449) 1:200, TCRβ (AB_466818) 1:200, GFP (AB_305643) 1:1000, NeuN (AB_177621) 1:200, GFAP (AB_305808) 1:500, Donkey-anti-rabbit AF647 (AB_10563288), Donkey-anti-rat AF647 (Invitrogen), Donkey-anti-goat Cy3 (Jackson Lab), anti hamster IgG (Biotin) (AB_2336137), Streptavidin-APC (Biozol). Pictures were taken with a laser scanning confocal microscope (Leica SP8).

### Data analysis

Statistical analysis was performed using two-way analysis of variances (ANOVA), followed by Bonferroni *posthoc* test if appropriate (version 6.0d, Prism software, GraphPad, USA). A value of p < 0.05 was considered significant.

## Results

### Generation of CB2-GFPTg mice

We integrated a green fluorescent protein (GFP) into a BAC clone spanning the Cnr2 locus (S2A Fig) and produced three positive founders with this transgene construct (CB2-GFPTg 4,12,13, S2B Fig). Through screening for GFP expression of PBMCs isolated from tail vein blood by flow cytometry, we identified one line (CB2-GFPTg 4) that exhibited reliable expression in the FITC channel (S2C Fig). Thus, subsequent analyses were carried out with that one line (CB2-GFPTg 4).

### GFP- and CB2-expression in CB2-GFPTg mice

Highest expression of GFP and CB2 mRNA was detected by quantitative real-time PCR (qPCR) in spleen tissue. Thymus showed a moderate expression of GFP and CB2 mRNA, whereas brain tissue did not show any expression (Fig 1C). Next, we analyzed GFP protein expression in these three organs using an anti-GFP antibody. In line with the qPCR results, we found highest protein expression in spleen, a moderate GFP expression in thymus but no GFP protein expression in brain tissue. GFP expression was not detectable in brain samples of Wt littermates (Fig 1D).

### Visualization of GFP in CB2-GFPTg mice

**Fig 2:**
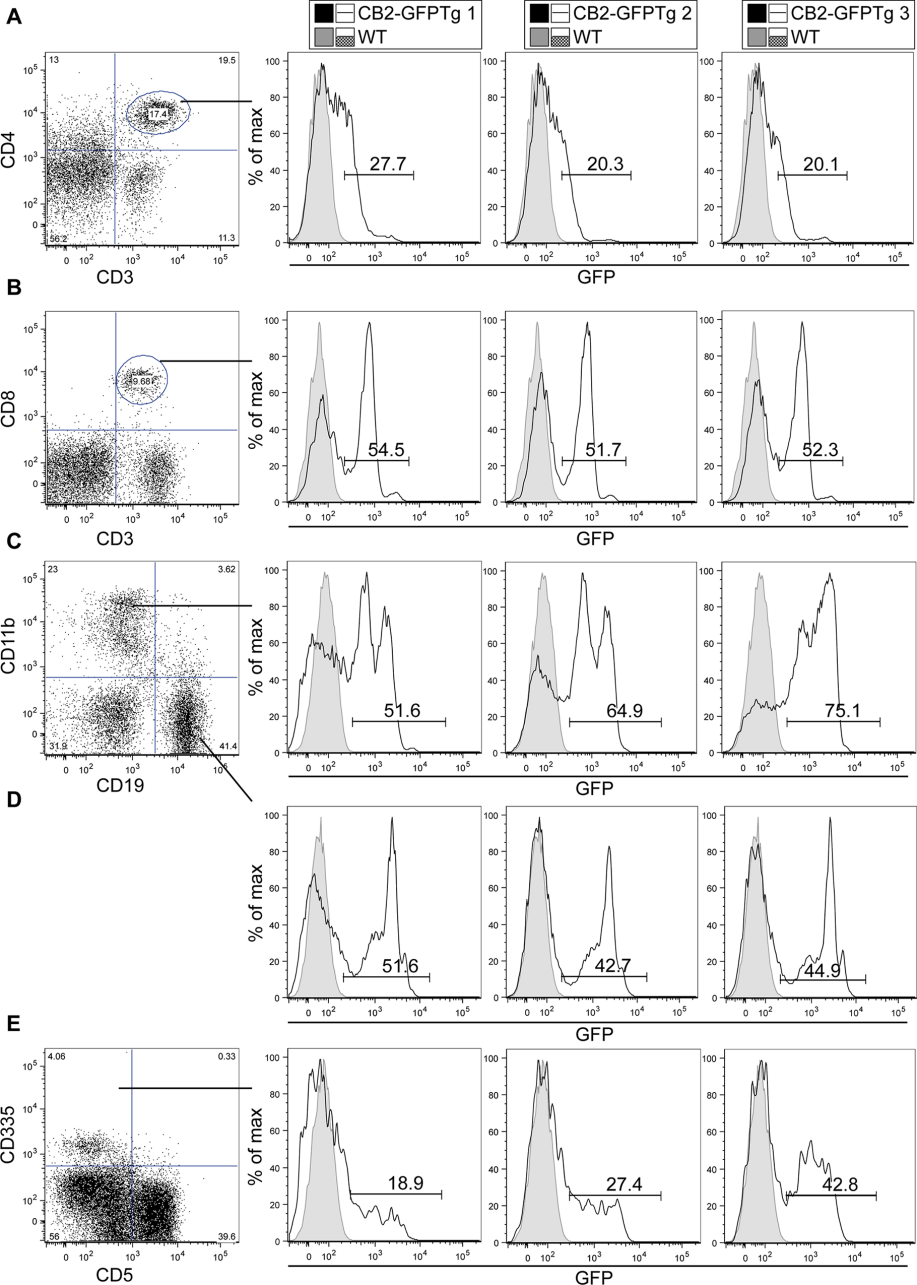
Comparison of GFP expression in murine PBMC using flow cytometry. Expression of CB2 protein was investigated using CB2-GFP mice and depicted by representative histograms of three individual transgenic mice in comparison to a non-transgenic littermate. In average 23% of CD4^+^ T-lymphocytes (a), 53% of CD8 T-lymphocytes (b), 64% of CD11b (c), 46% of CD19 (d) and 30% of CD335 cells (e) showed expression of GFP. All subsets were pre-gated on CD45+ cells and data analysis was performed using FlowJo software (Tree Star Inc., Ashland, USA).

Representative histograms of cellular subsets isolated from peripheral blood mononuclear cells (PBMCs) from transgenic and wildtype littermates (CB2-GFP founder 4) showed different expression profiles of GFP (Fig 2). CD45 positive cells were then stained for further cell surface markers representing various cell types in spleen. T-lymphocytes were identified by CD3 and CD4 or CD8 expression. In CD4^+^ lymphocytes, approximately 23% of the gated population showed a higher GFP expression (black line) compared to cells of non-transgenic littermates (shaded area in gray) (Fig 2A). In contrast to CD4^+^ lymphocytes, approximately 53% of all CD8^+^ lymphocytes exhibited strong expression of GFP (Fig 2B). We also found high GFP expression in CD11b^+^ cells (64%) (Fig 2C). However, this expression was heterogeneous, seen as two distinct peaks in the histograms, indicating a low and high GFP-expressing population in CD11b^+^ cells. B cells, identified by CD19, showed a homogenous expression of GFP in 46% of all cells (Fig 2D). We could also identify GFP expression in CD335 expressing natural killer cells (NK cells) (Fig 2E). However, expression level of CD335 itself differed between PBMC samples of CB2-GFPTg mice, indicating a high heterogeneity of CD335^+^ cell populations. Quantifying GFP expression in all different cellular subsets (S3 Fig), we found the highest expression of GFP in B cells (CD19^+^) and monocytes (CD11b^+^). Lymphocytes, CD4^+^ and CD8^+^, and NK-cells (CD335^+^) showed a significantly lower expression of GFP. In comparison to CB2-GFPTg mice, WT littermates showed no expression of GFP.

As we observed high expression of CB2 in the spleen (Fig 1C), it was of interest which cell types are expressing CB2 within the spleen. Thus, we performed immunohistochemical stainings for B cells (B220), T cells (TCRβ), macrophages (Iba1) and dendritic cells (DCs) (CD11c).

**Fig 3:**
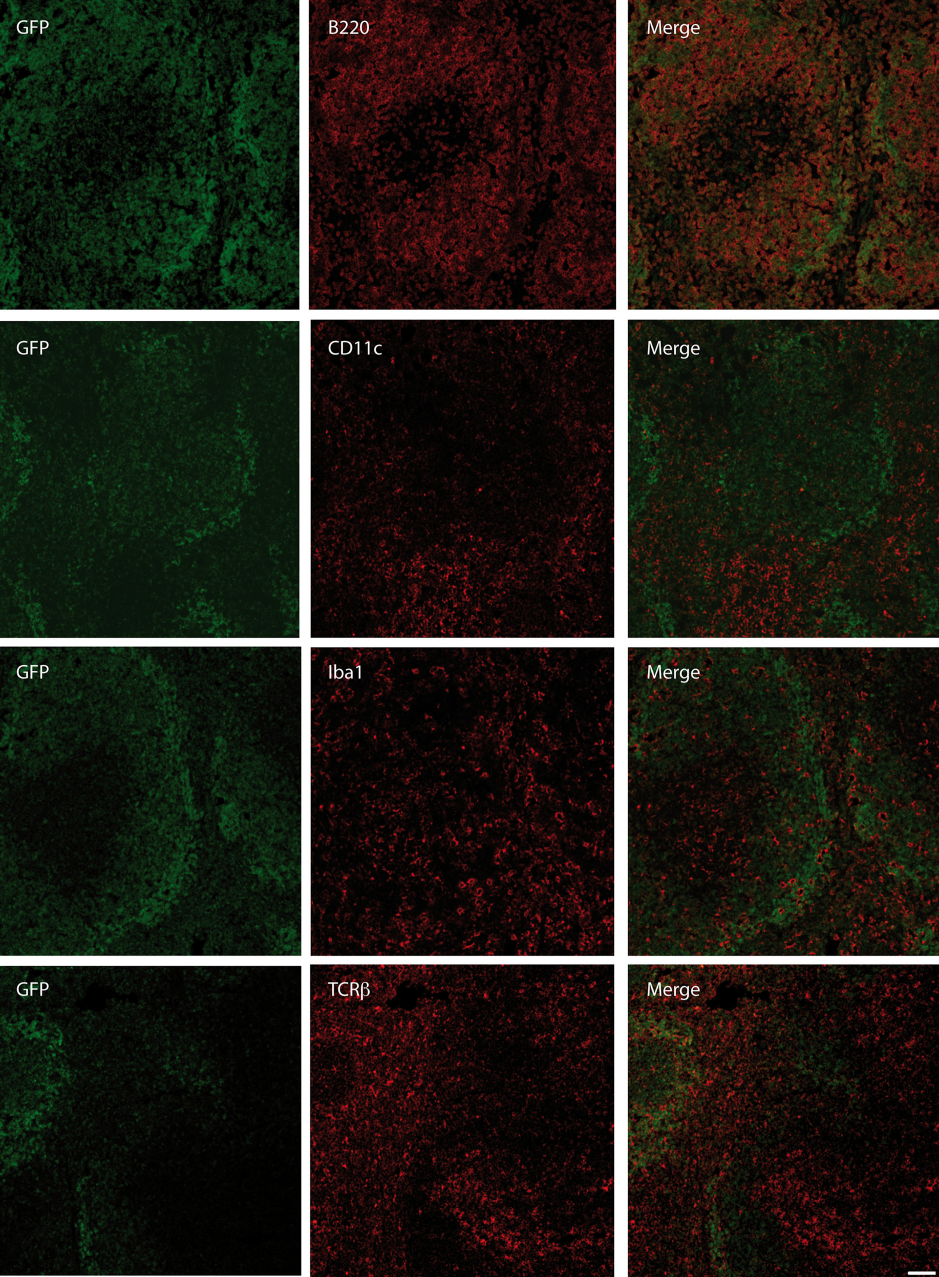
Analysis of CB2 expression in CB2-GFPTg mice. Immunohistochemical analysis of GFP expression in the spleen of CB2-GFPTg mice. Shown are representative spleen tissue sections of CB2-GFPTg stained for B220, CD11c, Iba1 and TCRβ (red), GFP expression is displayed as green staining. CB2-GFP is co-localized with B220 expression, whereas no co-expression with CD11c, Iba1 or TCRβ was observed (scale bar = 50 μm).

Immunohistochemical analysis showed a predominant expression of GFP in B cell follicles with a slight enrichment in the borders to the marginal zone (Fig 3). We detected a strong co-localization of GFP expression with B220, a marker mostly expressed by B cells. In contrast, we observed only faint co-expression of GFP with CD11c, Iba1 or TCRβ.

**Fig 4:**
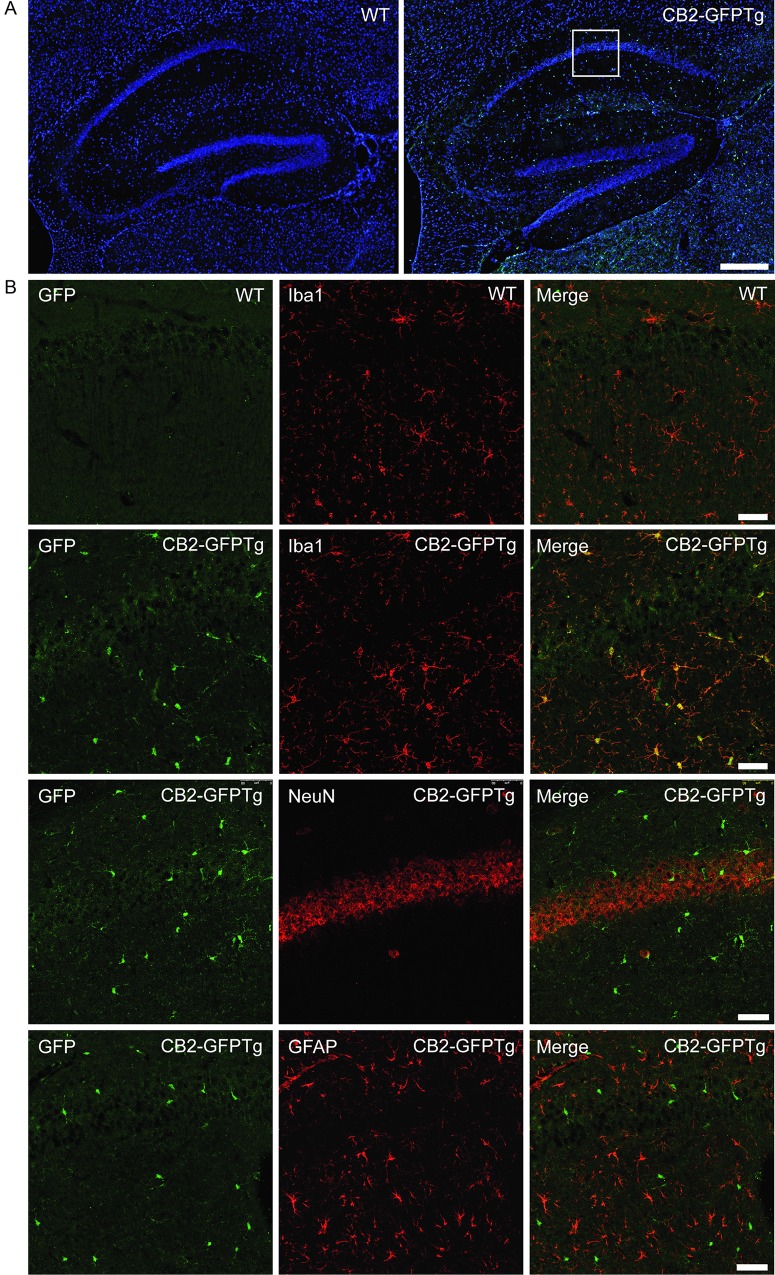
CB2-GFP expression in the hippocampus of WT and CB2-GFP mice. Immunohistochemical analysis of GFP expression in the brain of CB2-GFPTg mice. CB2-GFPtg mice show GFP expression in the hippocampus (a, right) which is not present in WT mice (a, left) (scale bar = 250 μm). CB2-GFP expression is co-localized with Iba1-staining (b, second row), but not with NeuN (b, third row) or GFAP (b, fourth row). Fig 4B, first row shows background analysis in WT mice (scale bar = 50 μm).

Beside CB2 expression in the spleen, we were also interested in CB2-GFP expression in the brain. Due to high autofluorescence of brain tissue in the GFP channel, immunohistochemical staining with an anti-GFP antibody was necessary to enhance the GFP signal. We observed CB2-GFP expression in the hippocampus (Fig 4A). Detailed analysis revealed that CB2-GFP co-localized with Iba1 expressing microglia cells (Fig 4B, second row) but not with NeuN positive neurons (Fig 4B, third row) or GFAP expressing astrocytes (Fig 4B, fourth row).

### Induction of GFP expression in CB2-GFPtg splenocytes

As our data revealed high expression of CB2 on splenic B cells, we wanted to test whether CB2 expression was regulated during pro-inflammatory stimulation. Thus, isolated splenocytes were stimulated *in vitro* with LPS or CpG and GFP expression was measured by flow cytometry.

**Fig 5:**
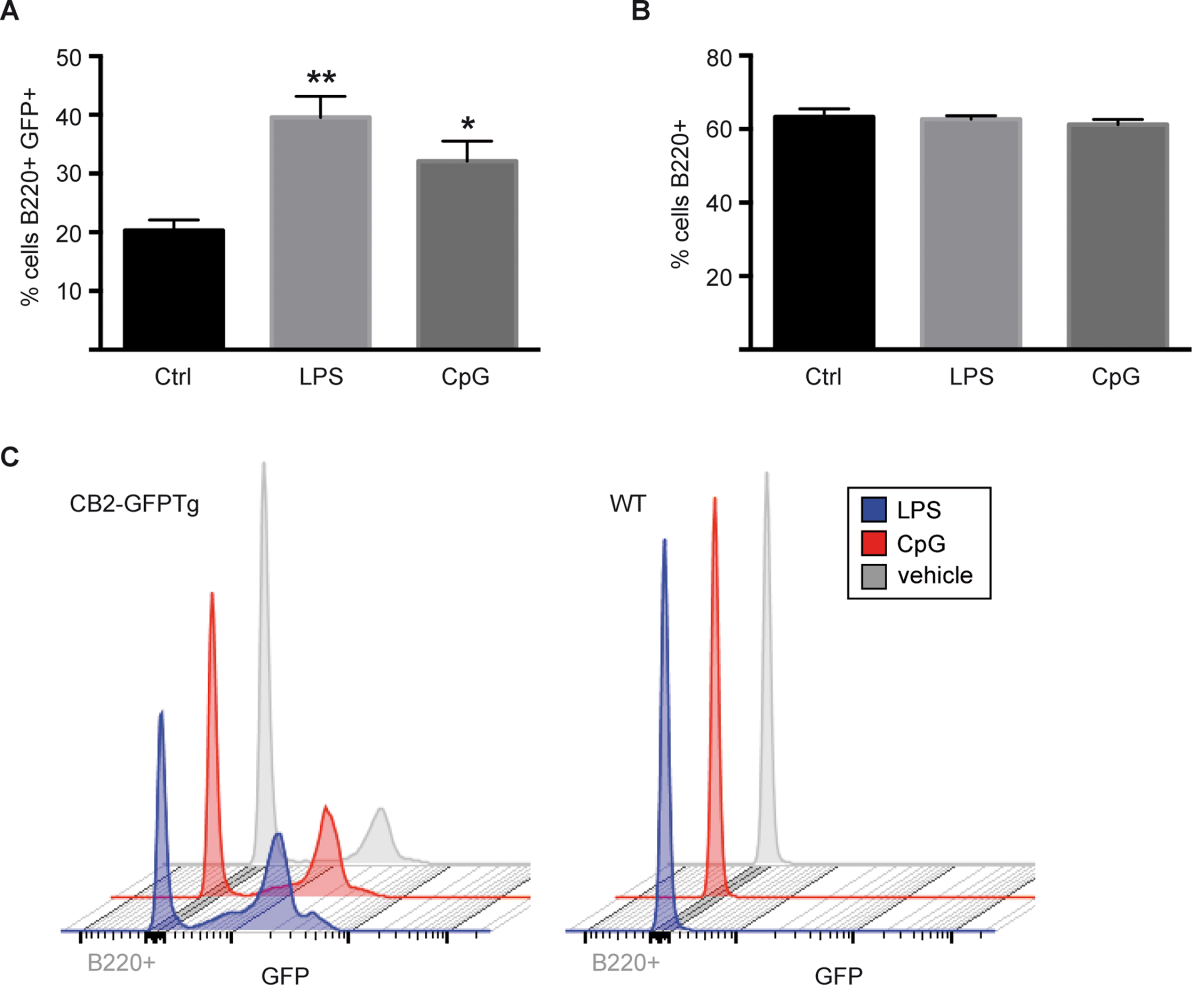
Enhanced GFP expression in stimulated splenic B cells. Splenocytes of WT and CB2-GFPTg mice were stimulated with LPS or CpG and GFP expression was analyzed by flow cytometry. GFP expression is significantly enhanced after stimulation in CB2-GFP mice (a,c) but not in WT mice (a). The number of B220^+^ cells is not altered by stimulation with LPS or CpG (b). N = 3, all samples were analyzed in triplicates. Data were analyzed by Students T-Test, *p < 0.05, **p < 0.001, to unstimulated genotype control.

Twenty percent of splenic B cells expressed GFP (Fig 5A). Following stimulation with LPS the number of CB2-GFP^+^ B cells significantly increased up to 39% (p < 0.01) and with CpG up to 32% (p < 0.05) (Fig 5A). In general, we noticed that the quantity of B220^+^ cells was not altered by stimulation with LPS or CpG (Fig 5B). We also observed that not the GFP expression intensity is increased by an inflammatory stimulus, but the number of GFP expressing cells in total is enhanced (Fig 5C).

## Discussion

Here, we report the generation of a novel BAC-based transgenic mouse line to study Cnr2 gene expression in mouse tissues. CB2-GFPTg mice express eGFP under the control of the endogenous CNR2-promotor. The use of this reporter mouse line permits the direct detection of CB2-expressing cells by flow cytometry, *Western* blot and histological analyses, thus avoiding usage of unspecific antibodies. Our data revealed GFP expression in all tissues where CB2 is expressed.

We first aimed to validate Cnr2 and GFP RNA expression and detected similar transcript levels in thymus and spleen. Highest CB2 and GFP expression levels were found in the spleen, a moderate expression in the thymus and negligible expression in brain tissue samples of CB2-GFPTg mice. This indicates that the locus of BAC-vector integration produces similar transcript amounts of GFP compared to Cnr2. Cnr2 mRNA in mouse lymphoid tissues have been studied previously by Schatz et al [14]. In line with our results, they demonstrated that Cnr2 was highly expressed in mouse spleen and to a lower extend in thymus using *Northern* blot analysis. However, whole brain tissue was devoid of any Cnr2 transcripts, although we cannot exclude Cnr2 expression in particular brain regions. We even substantiated this finding by analyzing protein expression using a GFP-specific *Western* blot of the same tissues. This expression pattern seems to be quite conserved, as CB2 mRNA expression in human lymphoid tissues resembles murine expression [15]. From this, we conclude that CB2-GFPTg mice could be used to identify CB2 expressing cells. When we analyzed GFP expression in blood cells, we found the highest expression levels in B cells and monocytes, lower expression levels in CD4 and CD8 lymphocytes and NK cells. Carayon and colleagues investigated CB2 expression levels in mononuclear cells isolated from peripheral human blood and could show that B lymphocytes, identified by CD20 expression, expressed the highest level of CB2 receptors, followed by NK cells, CD8^+^ and CD4^+^ lymphocytes [16]. These findings are in line with our results, showing the highest expression of CB2-GFP on B cells, followed by monocytes and NK-cells. Lowest expression of CB2 was found on lymphocytes (CD8^+^ > CD4^+^).

We further investigated the cellular distribution of the GFP fluorescence signal in the spleen. Immunohistochemical analysis of splenic tissue revealed a strong co-localization of CB2-GFP and B220, indicating an expression of CB2 on B cells within the spleen. On the contrary, we detected only a weak or no co-localization of CB2-GFP with Iba1, CD11c or TCRβ. Therefore, we conclude that CB2 is not or very faintly expressed on macrophages, dendritic cells or T cells under basal conditions in this tissue. Other groups already demonstrated that CB2 is mainly expressed on B cells in the spleen [17,18]. It was even found that B cells express highest levels of CB2 among immune cells [15,19]. Several studies indicate an important function for CB2 in B cell biology: Cannabinoids have been shown to promote proliferation and chemotaxis of B cells in vitro [20,21]. Additionally, *in vivo* studies revealed a relevant role for CB2 in B cell proliferation and maturation, which is specifically disturbed in CB2 deficient mice. These mice displayed reduced numbers of B cells in the marginal zone (MZ) [22,23]. In contrast, the number of macrophages, which are important for B cell development and retention [24,25], is not altered in CB2 deficient mice [26]. Another role for the CB2 receptor on B cells could be the regulation of B cell tropism for the splenic MZ, as Muppidi et al reported that CB2 acts as positional cue for MZ B cells and retains MZ B cells within the spleen [18]. To fully characterize CB2-GFP expression of our newly generated reporter mouse, we further analyzed brain tissue of CB2-GFPTg mice. Microglia, the resident immune cells of the brain, are known to express CB2. As microglia are present at high levels within the CA1-region of the hippocampus, we focused on CB2-GFP expression in hippocampal tissue. Here, we observed CB2-GFP expression on microglia of naïve CB2-GFP mice. The expression level, however, is dependent on their state of activation [27–30]. CB2 can modulate microglia migration and infiltration to sides of inflammation within the CNS [31–33]. It is described that microglia do not express CB2 in healthy tissue [28]. However, CB2 expression on microglia within the context of several neuro-inflammatory diseases, such as Alzheimer’s or Huntington’s Disease, simian immunodeficiency virus-induced encephalitis, HIV encephalitis, and multiple sclerosis was shown by several publications [34–37]. Our results indicate that CB2 is expressed on microglia even under non-inflammatory conditions. Recent publications also detected CB2 expression on neurons [38], however, we were not able to reveal these findings in our experimental conditions. Therefore, the debatable finding of CB2 expressing neurons has to be proven in further studies.

To study the influence of inflammatory stimuli on the regulation of CB2 expression, we stimulated isolated splenocytes with LPS or CpG and analyzed the expression of CB2-GFP by flow cytometry. Our data demonstrate that stimulation with both LPS and CpG causes a significant upregulation of CB2-GFP. This is in contrast to the report by Lee et al., who observed reduced CB2 expression on B cells as a consequence of LPS-stimulation [39]. This contrasting result might be explained by the fact that they quantified CB2 expression on mRNA levels using a densitometric analysis, while we quantified GFP expression as a reporter for CB2 expression on protein level.

However, as the total number of large B220^+^ is not changed after stimulation with LPS or CpG (data not shown), treatment of splenocytes with a pro-inflammatory stimulus does not seem to influence B cell proliferation after short-time LPS-incubation. Another group already reported that LPS-mediated B cell proliferation is time- and dose-dependent [40]. This controversial result might be explained by the difference in stimulation time as Xu et al observed proliferation after 48h of LPS-stimulation. We chose a shorter duration of stimulation based on the fact that the GFP signal rapidly decreases in culture and is not longer detectable after 24h of splenocyte cultivation.

In summary, we provide here a new mouse model to study the expression and regulation of CB2 receptors in different organs. Since commercially available mouse-specific antibodies raised against CB2 produce ambiguous and vague results, this is, to our knowledge, the first model to study CB2 protein expression using GFP as a corresponding reporter. This will provide new insights into the regulation of this molecule upon different inflammatory conditions.

## Supporting Information

S1 Fig Cloning strategy for the generation of the CB2/eGFP-FRT-Neo® fragment. Explanation in the main text.
(TIF)

S2 Fig Southern blot strategy for the CB2-GFPTg mice. (a) Schematic representation of the endogenous Cnr2 and CB2-GFP BAC locus. Restriction sites and the genomic probe (black bar) used for *Southern blot* analysis are indicated. Exons are represented as rectangles; an FRT site as triangle. (b) *Southern* blot analysis of Wt and CB2-GFP mice. Founder and Wt genomic DNA were double digested with PstI/AseI. A genomic PstI fragment of 8,5kb corresponds to the Wt fragment, whereas the transgenic mice display an additional AseI site which results in a shorter fragment of 3,3 kb. Restriction enzymes. P: *Pst*I; A: *Ase*I; C: *Cla*I. (c) GFP expression in founder 4 Wt and transgenic offsprings. Data shows CB2-GFP expression of three individual animals compared to Wt littermate. Only number of CB2-GFP expressing cells but not intensity of the GFP signal is altered in founder 4 offsprings.
(TIF)

S3 Fig Quantitative analysis of CB2-GFP expression on PBMCs. Bar graphs show mean fluorescence intensity (MFI) of GFP expression measured by flow cytometry using the FITC channel. Cells were pregated on live-cells and CD45 expression. Highest expression of GFP was found in CD11b^+^ monocytes and CD19^+^ B cells. A significant reduction in GFP expression was found in T-cells (CD8^+^ > CD4^+^) and NK cells compared to B-cells and monocytes. Statistical analysis was performed using two-way analysis of variances (ANOVA), followed by Bonferroni *posthoc* test (version 6.0d, Prism software, GraphPad, USA). A value of p < 0.05 was considered significant.
(TIF)

S1 Table Primer sequences used of the generation of the CB2-GFP BAC and for the amplification the *Southern* blot probe. (DOCX)
