# Correction: Expression Analysis of CB2-GFP BAC Transgenic Mice

**DOI:** 10.1371/journal.pone.0145472

**Published:** 2015-12-17

**Authors:** Anne-Caroline Schmöle, Ramona Lundt, Benjamin Gennequin, Hanna Schrage, Eva Beins, Alexandra Krämer, Till Zimmer, Andreas Limmer, Andreas Zimmer, David-Marian Otte

There are multiple errors in S1 Table. Please view the correct S1 Table below.

## Supporting Information

S1 Table Primer sequences used of the generation of the CB2-GFP BAC and for the amplification the *Southern* blot probe. (DOCX)
