# Supplementary figures and images for: Expression Analysis of CB2-GFP BAC Transgenic Mice
Source: PLoS One. 2015 Sep 25;10(9):e0138986. doi: 10.1371/journal.pone.0138986 (PMC4583291; doi:10.1371/journal.pone.0138986)

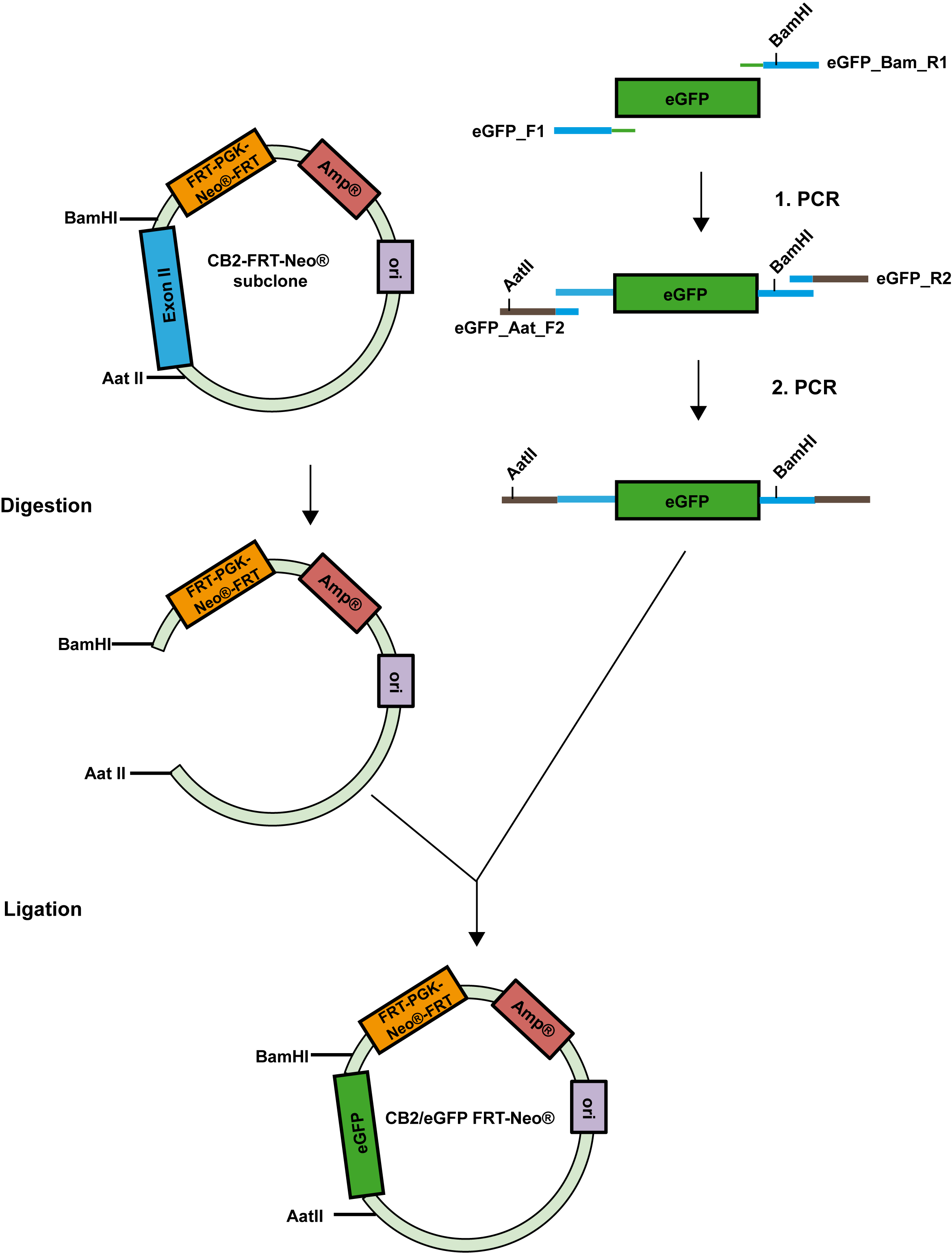

Supplement: S1 Fig — Explanation in the main text. (TIF) [file pone.0138986.s001.tif]

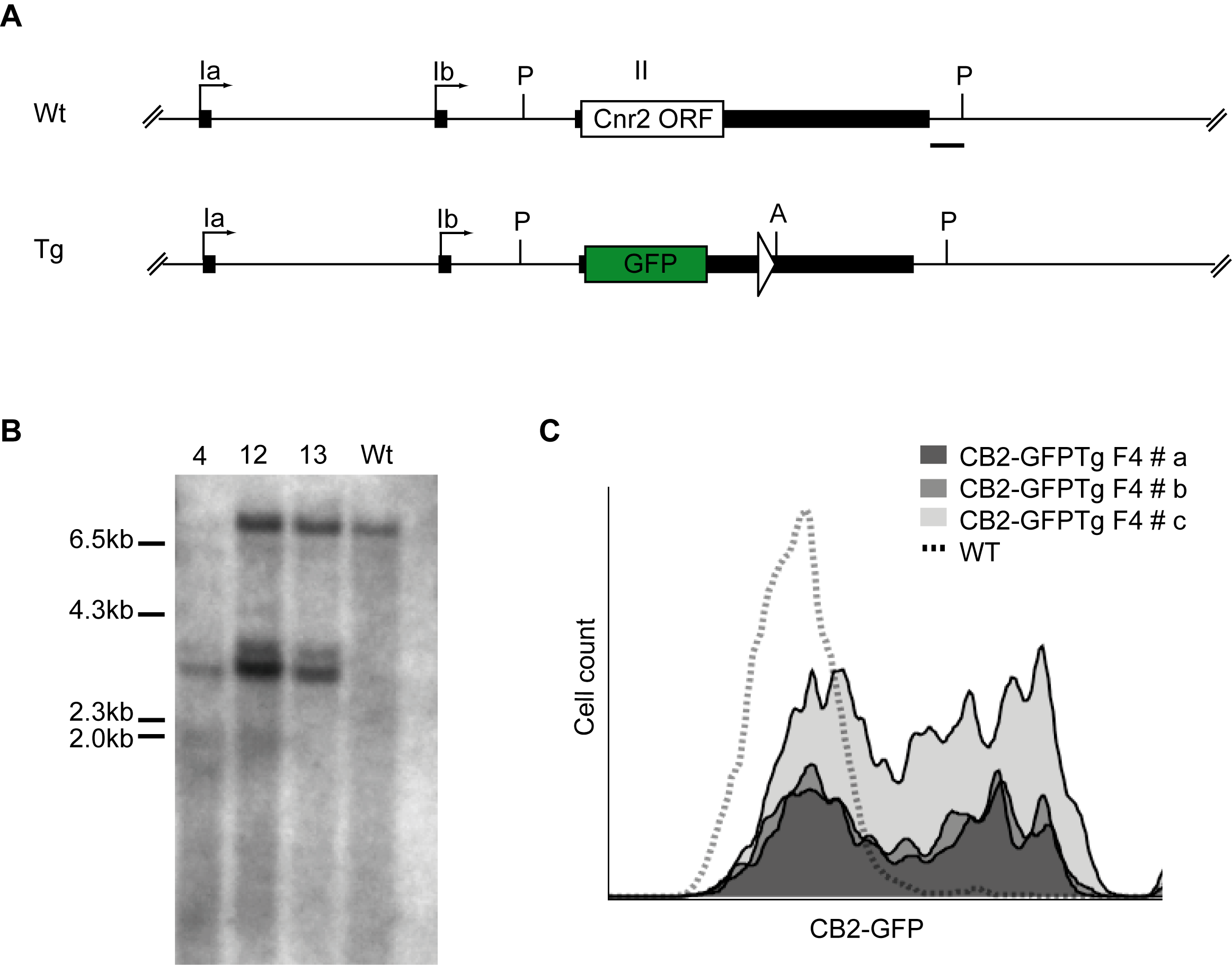

Supplement: S2 Fig — (a) Schematic representation of the endogenous Cnr2 and CB2-GFP BAC locus. Restriction sites and the genomic probe (black bar) used for Southern blot analysis are indicated. Exons are represented as rectangles; an FRT site as triangle. (b) Southern blot analysis of Wt and CB2-GFP mice. Founder and Wt genomic DNA were double digested with PstI/AseI. A genomic PstI fragment of 8,5kb corresponds to the Wt fragment, whereas the transgenic mice display an additional AseI site which results in a shorter fragment of 3,3 kb. Restriction enzymes. P: PstI; A: AseI; C: ClaI. (c) GFP expression in founder 4 Wt and transgenic offsprings. Data shows CB2-GFP expression of three individual animals compared to Wt littermate. Only number of CB2-GFP expressing cells but not intensity of the GFP signal is altered in founder 4 offsprings. (TIF) [file pone.0138986.s002.tif]

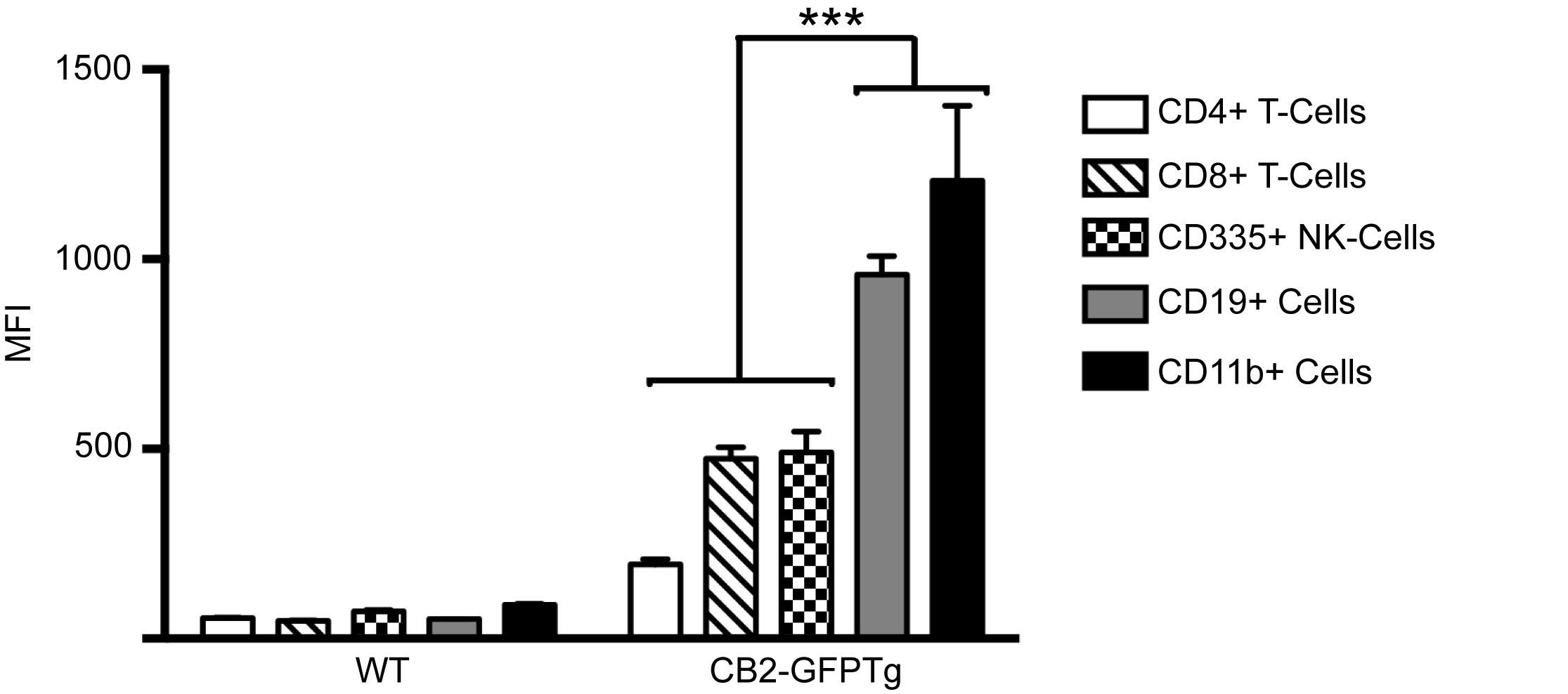

Supplement: S3 Fig — Bar graphs show mean fluorescence intensity (MFI) of GFP expression measured by flow cytometry using the FITC channel. Cells were pregated on live-cells and CD45 expression. Highest expression of GFP was found in CD11b+ monocytes and CD19+ B cells. A significant reduction in GFP expression was found in T-cells (CD8+ > CD4+) and NK cells compared to B-cells and monocytes. Statistical analysis was performed using two-way analysis of variances (ANOVA), followed by Bonferroni posthoc test (version 6.0d, Prism software, GraphPad, USA). A value of p < 0.05 was considered significant. (TIF) [file pone.0138986.s003.tif]
