# Supplementary material for: Correction: Expression Analysis of CB2-GFP BAC Transgenic Mice
Source: PLoS One. 2015 Dec 17;10(12):e0145472. doi: 10.1371/journal.pone.0145472 (PMC4683073; doi:10.1371/journal.pone.0145472)
Supplement: S1 Table — (DOCX) [file pone.0145472.s001.docx]

Supplementary Material

Tab.S1

| CB2subF | 5`-GTGCCTTCTAGAAGACCTATTTCTAGGGCAAGGATGTGTTTGATGTATAAC  AATTGACAGCTTGTCTGTAAGCGGATG-3` |
| --- | --- |
| CB2subR | 5`-GTTTAAGTTCCTCGGAGTTCTTGTTAAGTTCAACGGACAAAAGATAGACTC  AATTGGCTCTCCTGAGTAGGACAAATC-3` |
| FRTneoF | 5`-CACTATCCCAGTCTTGCCTAGCTTCAGTACAAAGCAAGATATTCACACAGA  ATTAACCCTCACTAAAGGGCG-3` |
| FRTneoR | 5`-CAGTGGGGCTGAGTCAAGCATCACTGGGAACAGCCTAGGACCCAAAGAAT  TAATACGACTCACTATAGGGCTC-3` |
| eGFP_F1 | 5`-acagcccagtcttctgggacagctccagtagaagaagccaaagcccatcc  atggtgagcaagggcgaggagctgt-3` |
| eGFP_Bam_R1 | 5`-gacaggtggtgtcagcagttggagcagcctggagttctggatcctggctc  ttacttgtacagctcgtccatgccg-3` |
| eGFP_*Aat_*F2 | 5`-GCTCTCAGTTGACGTCATCACCTGTTAACATTCAAGGATTGTTTTCTCCTTG  CCCACAGCCCAGTCTTCTGGGACAGCT-3` |
| eGFP_R2 | 5`-CTCTTCGAGGGAGTGAACTGAACGGACTTCTGACTCGGGCTGTTTCCAGTA  GAAAGACAGGTGGTGTCAGCAGTTGGAG-3` |
| Probe F | 5´- TCTCCCAGTTTTCCCCACAC-3´ |
| Probe R | 5´- GAGTAACGATTGGCTTGGAAGG-3´ |
